# SIRT3 activator Honokiol attenuates β-Amyloid by modulating amyloidogenic pathway

**DOI:** 10.1371/journal.pone.0190350

**Published:** 2018-01-11

**Authors:** Sindhu Ramesh, Manoj Govindarajulu, Tyler Lynd, Gwyneth Briggs, Danielle Adamek, Ellery Jones, Jake Heiner, Mohammed Majrashi, Timothy Moore, Rajesh Amin, Vishnu Suppiramaniam, Muralikrishnan Dhanasekaran

**Affiliations:** Department of Drug Discovery and Development, Harrison School of Pharmacy, Auburn University, Auburn, AL, United States of America; Torrey Pines Institute for Molecular Studies, UNITED STATES

## Abstract

Honokiol (poly-phenolic lignan from *Magnolia grandiflora*) is a Sirtuin-3 (SIRT3) activator which exhibit antioxidant activity and augment mitochondrial functions in several experimental models. Modern evidence suggests the critical role of SIRT3 in the progression of several metabolic and neurodegenerative diseases. Amyloid beta (Aβ), the precursor to extracellular senile plaques, accumulates in the brains of patients with Alzheimer’s disease (AD) and is related to the development of cognitive impairment and neuronal cell death. Aβ is generated from amyloid-β precursor protein (APP) through sequential cleavages, first by β-secretase and then by γ-secretase. Drugs modulating this pathway are believed to be one of the most promising strategies for AD treatment. In the present study, we found that Honokiol significantly enhanced SIRT3 expression, reduced reactive oxygen species generation and lipid peroxidation, enhanced antioxidant activities, and mitochondrial function thereby reducing Aβ and sAPPβ levels in Chinese Hamster Ovarian (CHO) cells (carrying the amyloid precursor protein-APP and Presenilin PS1 mutation). Mechanistic studies revealed that Honokiol affects neither protein levels of APP nor α-secretase activity. In contrast, Honokiol increased the expression of AMPK, CREB, and PGC-1α, thereby inhibiting β-secretase activity leading to reduced Aβ levels. These results suggest that Honokiol is an activator of SIRT3 capable of improving antioxidant activity, mitochondrial energy regulation, while decreasing Aβ, thereby indicating it to be a lead compound for AD drug development.

## Introduction

Alzheimer’s disease is a neurodegenerative disease characterized by a decline in cognition due to morphological and functional alterations to neurons. Pathologically, it is characterized by abnormal accumulation of extracellular senile plaques consisting of amyloid beta (Aβ), and intracellular neurofibrillary tangles consisting of hyperphosphorylated tau protein [1]. Epidemiological evidence shows that patients with type 2 diabetes mellitus have an increased risk of developing Alzheimer’s disease. This can be attributed to altered glucose metabolism, impaired insulin signaling, and insulin resistance [2–4]. Insulin resistance (IR) results in reduced glucose uptake and utilization, which compromises cell energy and homeostatic functions, thereby promoting oxidative stress and mitochondrial dysfunction. This energy deficiency results in the disruption of the neuronal cytoskeleton and synaptic connection [5,6]. Interestingly, brain insulin resistance has been known to accelerate the accumulation of Aβ and plaque formation in the brain by enhancing amyloidogenic processing of the amyloid precursor protein [7]. In addition, high insulin levels tend to inhibit Aβ degradation, thereby increasing amyloid accumulation which leads to neurodegeneration and irreversible cognitive dysfunction [8].

Mitochondria play a crucial role in the normal functioning of neurons and synapses by supplying constant energy in the form of ATP. Deficits in energy metabolism lead to increased oxidative stress and endoplasmic reticulum stress thereby promoting mitochondrial dysfunction. Oxidative stress results in the generation of excessive reactive oxygen species (ROS) and reactive nitrogen species (RNS) [9–12], which promotes the formation of lipid peroxides and damages the RNA, DNA, and proteins. Moreover, ROS can up regulate the expression of APP, β and γ-secretase to generate Aβ deposition, and fibrilization [5, 13, 14]. This Aβ in turn interacts with various mitochondrial proteins, disrupting the electron transport chain and increasing reactive oxygen species, thereby decreasing the levels of ATP [15–17]. Therefore, oxidative stress and mitochondrial dysfunction may be significantly implicated in the development and progression of Alzheimer’s disease [18].

Sirtuins are a family of proteins that act predominantly as nicotinamide adenine dinucleotide (NAD)-dependent deacetylases, causing post-translational modifications in target proteins to regulate their function. Seven sirtuin family members exist, out of which SIRT3, SIRT4, and SIRT5 localize exclusively within mitochondria while the reminder of the sirtuins are localized within the cytoplasm and nucleus [19]. Acetylation causes proteins required for proper mitochondrial function to malfunction which leads to oxidative stress. These abnormalities are prevented by SIRT3 due to its deacetylating properties [20–23]. In addition, SIRT3 has been shown to act as a pro-survival factor that plays an essential role in protecting neurons experiencing excitotoxicity [24]. Until recently, the only means to achieve high intracellular levels of SIRT3 was through calorie restriction and endurance exercise [25–28]. However, Honokiol [2-(4-hydroxy-3-prop-2-enyl-phenyl)-4-prop-2-enyl-phenol] has recently been considered to be a pharmacological activator of SIRT3 and known to modulate the pathologies of AD [29]. Honokiol, by binding to SIRT3 causes increased expression of AMPK-5' adenosine monophosphate-activated protein kinase, which plays a crucial role in cellular energy homeostasis. Additionally, it is known to increase the expression of PGC-1α. Furthermore, SIRT3 is a downstream target gene of PGC-1α and SIRT3 mediates the PGC-1α effects on cellular ROS production and mitochondrial biogenesis [30]. Honokiol has also been shown to possess non-adipogenic partial PPAR-γ agonistic activity [31]. PPAR-γ activity is known to promote glucose and lipid metabolism, oxidative phosphorylation, and mitochondrial biogenesis by increasing the expression of PGC-1α, a master regulator of mitochondrial biogenesis [32, 33]. PGC-1α decreases Aβ generation and increases non-amyloidogenic sAPPα levels by reducing the β-APP cleaving enzyme (BACE1 or β-secretase) gene transcription via PPAR-γ-dependent mechanism and directly through SIRT3 [34, 35].

Aβ is a proteolytic product of the amyloid-β precursor protein (APP) and is generated through sequential cleavages by enzymes called β- and γ-secretases. During this amyloidogenic processing, β-secretase first cleaves the type I transmembrane APP protein to generate an extracellular fragment known as sAPPβ and a membrane-associated carboxyl terminal fragment known as APP β-CTF. APP β-CTF is then cleaved by γ-secretase to release Aβ. Alternatively, APP can be subjected to a non-amyloidogenic processing and cleaved by α-secretase within the Aβ domain. α-secretase-mediated cleavage precludes Aβ generation and generates an extracellular domain of APP known as sAPPα instead [36,37]. β-cleavage of APP is the first and rate-limiting step in Aβ production. The transmembranous aspartic protease β-site APP cleaving enzyme 1 (BACE1) has been identified as the essential β-secretase in vivo [38]. The level and activity of BACE1 are found to be elevated in postmortem brain of sporadic AD patients [39, 40], suggesting a causative role of BACE1 in AD.

In this study, we hypothesize that Honokiol, a SIRT3 activator suppress oxidative stress, enhance mitochondrial functions and modulate Aβ levels by inhibiting BACE1 activity. PS70 cell lines (Chinese Hamster Ovarian cells expressing Swedish mutant APP (APPswe) and wild type human PSEN1 were used in this study. The swedish mutant APP (APPswe) has been shown to induce early AD-like histopathology with dispersed deposits of Aβ and aberrant tau protein expression [41, 42]. The PSEN1 gene and its protein are part of the γ-secretase complex which play a crucial role in processing APP and is known to increase Aβ levels [43]. The net effect of these two genes is increased secretion of Aβ by the cell which aids in studying the effect of Honokiol on the amyloidogenic pathway. Various other studies have employed APP-CHO cells to study and validate the amyloidogenic pathway [44–46]. Therefore, this study aimed at elucidating the molecular mechanisms and signaling pathways by which Honokiol modulate Aβ levels in PS70 cells.

## Materials and methods

### Cell culture

PS70 cell lines (Chinese Hamster Ovary cells–CHO expressing Swedish mutant APP (APPswe) and wild type human PSEN1) was a kind gift from Dr. Raj Amin. Cells were grown in DMEM (VWR, USA) supplemented with 10% fetal bovine serum (FBS; Biosciences, USA), 100U/ml penicillin (Corning, USA) and 100μg/ml streptomycin (Corning, USA) in a humidified atmosphere of 5% CO2/95% air at 37°C. The cells were cultured in the presence of G418 (200 μg/ml, Invitrogen) and puromycin (7.5 μg/ml, ThermoFischer Scientific) to maintain selection for the expression plasmid. The cells were plated at an appropriate density according to each experimental scale.

### Treatment strategies

Honokiol was purchased from Cayman chemicals, USA. Regarding the cell viability assay, different doses of Honokiol (0.5, 1, 2, 5, 10 and 20μM) were incubated with PS70 cells for 2 different times (24 and 48 hours) periods in the presence of insulin 10nM and serum. However, based on cell viability results and to elucidate the molecular mechanisms of action, PS70 cells were treated with Honokiol (5 and 10μM) for 24 and 48 hours. Insulin (10nM) was used as a positive control. To establish insulin resistance (IR) in PS70 cells, we used high concentrations of insulin (10nM) for both 24 and 48 hours [47]. Insulin-degrading enzyme (IDE) is involved in clearance of Aβ in the brain as both insulin and Aβ are catabolized by IDE [48]. In presence of high insulin, IDE is diverted to degrade insulin, consequently allowing APP-Aβ accumulation [49, 50]. IDE is thought to be a link connecting hyperinsulemia, IR, and AD [51, 52].

### Cell viability assay

PS70 cells were seeded in 96-well plates with 1000 cells/well in culture medium and following their fixation, cells were treated with Honokiol (0.5, 1, 2, 5, 10 and 20μM concentrations) for 24 and 48 hours. Cell viability was assessed using the PrestoBlue® assay (Invitrogen, Carlsbad, CA, USA) according to manufacturer's instructions. Absorbance was measured by a spectrophotometer (BioTek, Winooski, VT, USA). The results were evaluated as percent of control and calculated as mean±SEM. Furthermore, microscopic imaging was performed on the PS70 cells to validate the cell viability and study the morphological changes seen at various concentrations.

### Determination of ROS generation

Reactive oxygen species generation was estimated spectrofluorometerically by conversion of the non-fluorescent chloromethyl-DCF-DA (2′,7′-dichlorofluorescin diacetate, DCF-DA, VWR, USA) to fluorescent DCF at an excitation wavelength of 492 nm and an emission wavelength of 527 nm. The generation of ROS was measured, normalized to total protein content and reported as relative fluorescence intensity/mg protein. The fluorimetric reading was measured with BioTek Synergy HT plate reader (BioTek, VT, USA). Results were expressed as percentage change from the control [53].

### Measurement of mitochondrial ROS levels

Mitochondrial ROS levels were measured using mitochondrial specific dihydro- 244 rhodamine (DHR) indicator purchased from Biotium. DHR is an uncharged non-fluorescent ROS indicator that accumulates in the mitochondria and becomes oxidized to the cationic rhodamine123, which exhibits a green fluorescence. The PS70 cell lines were stained according to the manufacture’s protocol. Fluorescence was measured using a multispectral-fluorescent plate reader (Bio-Tek) at excitation/emission wavelengths (λEx/ λEm) at 505/ 534 nm. [54].

### Estimation of lipid peroxidation

Colorimetric assay procedure using thiobarbituric acid was used to quantify the lipid peroxide content. The index of lipid peroxidation was estimated by measuring the malondialdehyde (MDA) content in the form of thiobarbituric acid reactive substances (TBARS). TBARS was measured in the plate reader at 532 nm and calculated as TBARS formed per mg protein. Results were expressed as percentage change from the control [55, 56].

### Assay of superoxide dismutase (SOD) activity

SOD activity was measured spectrophotometrically following the Marklund and Marklund method, using pyrogallol as substrate at 420 nm. Results were expressed as percentage control [57].

### Estimation of catalase activity

Catalase activity was determined spectrophotometrically where the degradation of hydrogen peroxide is measured at 240 nm [58, 59]. Results were expressed as percentage control.

### Glutathione peroxidase assay

Spectrophotometric estimation of glutathione peroxidase was performed according to the method of Lawrence and Burk [60]. The activity was calculated as glutathione (μmol) oxidized/mg total protein.

### Glutathione reductase activity

Glutathione Reductase assay was performed spectrophotometrically using glutathione reductase assay kit (Cayman Chemicals, no. 703202). Values were based on a standard curve and calibrated to the total levels of protein concentrations.

### Mitochondrial Complex-I activity

Mitochondrial Complex-I activity (NADH dehydrogenase activity) was assessed based on the NADH oxidation. Oxidation of NADH by the NADH dehydrogenase was measured spectrophotometrically at 340nm. Results were expressed as percentage control [61].

### Mitochondrial Complex-IV activity

Complex-IV activity was based on the Cytochrome-c oxidation. The Cytochrome-C oxidation was measured spectrophotometrically at 550 nm and the Complex IV activity was expressed as cytochrome-C oxidized/mg protein [62].

### Mitochondrial membrane potential assay

The mitochondrial membrane potential through microplate assay was measured in 96 well plate utilizing tetramethylrhodamine ethyl ester (TMRE) according to the manufacturer’s instructions (TMRE; Biotium, no.70016). TMRE florescent intensity (Ex: 549nm, Em: 575nm) was measured by a BioTek Synergy HT plate reader (BioTek, VT, USA). The results were expressed as percentage change from the control. In addition, imaging of the mitochondrial membrane potential was evaluated using fluorescence microscope with the fluorescent dye tetramethylrhodamine methyl ester TMR.

### Western blot analysis

Conditioned media from treated cells were assayed for sAPPα, sAPPβ and secreted Aβ by Western blot. PS70cells were lysed in RIPA buffer (Roche, USA) and equal protein amounts of cell lysates were analyzed by Western blot. Each sample was denatured at 95°C for 5minutes before loading onto freshly prepared 10% SDS-PAGE gel for protein separation. Separated proteins on SDS-PAGE were transferred onto polyvinylidene fluoride membrane. Non-specific binding sites on the membranes were blocked with 5% fat-free milk in Tris-buffered saline plus 0.1% Tween-20 (TBST) at pH 7.4. The membranes were incubated overnight at 4°C with specific antibody constituted in 5% BSA in TBST. Primary antibodies used in this study included: AMPK (#2532), phospho-AMPK Thr172 (#2535), CREB (#4820) from CST; Anti-SIRT3 antibody (ab86671), Anti-PGC1α (ab54481), Anti-beta Amyloid 1–42 antibody (ab12267), Anti-beta Amyloid 1–40 antibody [BDI350] (ab20068), Anti-beta Actin antibody (ab8227), Anti-GAPDH (ab8245), Anti-ADAM10 antibody [EPR5622] (ab124695) from abcam; APP C-terminal antibody pAb751/770 (EMD Biosciences, La Jolla, CA, USA); Anti-BACE1 monoclonal antibody (MAB5308), anti-ADAM10 polyclonal antibody and Anti phospho-CREB (pAb06-519) from Merck Millipore; 6E10 (against sAPPα and β-CTF) and anti-sAPPβ antibodies from Covance. Membranes were then washed with TBST (3X, each for 10 min) and incubated with species dependent Goat Anti-Rabbit (H+L) IgG DyLight550 conjugated secondary antibodies (Invitrogen™) for 60 min at room temperature. Membranes were again washed three times for 10 minutes with TBST after incubation with each antibody. After washing, membranes were analyzed in FluorChem® system Imaging. Band densities for each sample were normalized to their respective β-actin or GAPDH signal and reported as percentage control.

### α-secretase activity assay

The activity of α-secretase in cells was measured by using InnoZyme TACE Activity Kit (Millipore), following the manufacturer’s protocols.

### β-secretase activity assay

β-site-APP cleaving enzyme (BACE) or β-Secretase activity was determined fluorimetrically with a commercially available β- Secretase activity kit (Biovison, California, USA) according to the manufacturer’s instructions. Beta-secretase activity was represented as relative fluorescence unit per mg of total protein.

### Aβ ELISA assay

After treatment, conditioned media from the treated and untreated cells were collected to detect secreted Aβ1–40 and Aβ1–42. The Aβ1–40 and Aβ1–42 concentrations were quantified using ELISA kits following the manufacturer's protocol. The optical densities of each well at 450 nm were read on a microplate reader (Biotek FLx800, USA)] and the sample Aβ1–40 and Aβ1–42 concentrations were determined by comparison with the Aβ1–40 and Aβ1–42 standard curves. All readings were in the linear range of the assay.

### Protein estimation

Protein quantification was determined using the Thermo Scientific Pierce 660 nm Protein Assay reagent kit (Pierce, Rockford, IL).

### Statistical analysis

All data are expressed as means ± SEM. Statistical analyses were performed using one-way analysis of variance (ANOVA) followed by an appropriate post-hoc test including Tukey's and Dunnett's method (*p*< 0.05 was considered to indicate statistical significance). All statistical analyses were performed using the Prism-V software (La Jolla, CA, USA).

## Results

### Effect of Honokiol on PS70 cell viability

**Fig 1:**
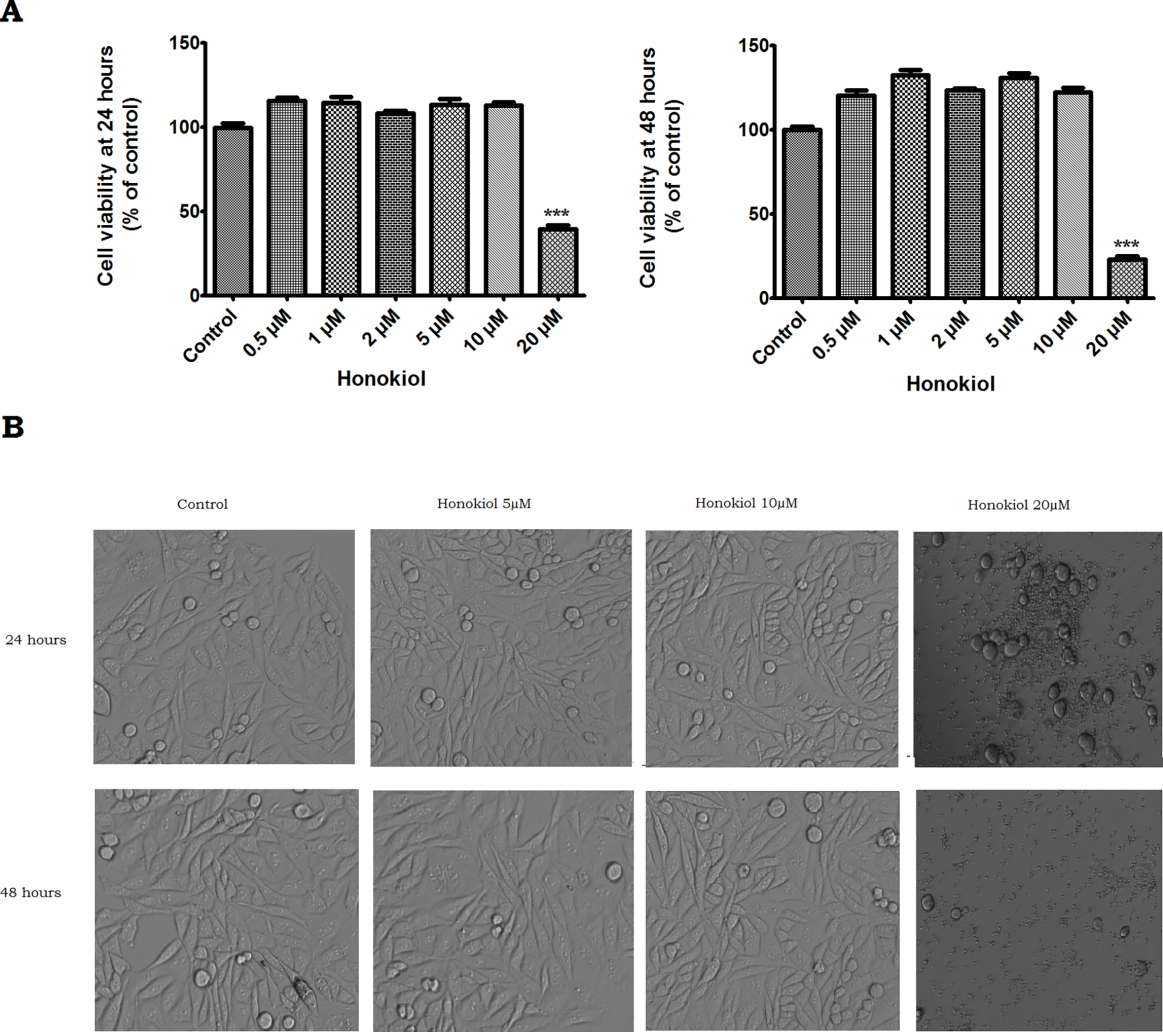
Effect of honokiol on PS70 cells. (A) PS70 cells were treated with various concentrations of honokiol (0, 1, 2, 5, 10 and 20μM) for 24 hours and 48 hours and then analyzed by Prestoblue cell viability assay. DMSO (0.1%) was used as the vehicle for honokiol. Data are expressed in terms of percent of control cells (non-honokiol-treated) as the means ± SE. ***P< 0.001 vs. vehicle-treated (control) cells. (B) Morphological changes in PS70 cells at 24h and 48 h following treatment with Honokiol (0, 1, 2, 5, 10 and 20μM). Scale bar = 100μm.

The effect of Honokiol treatment on the PS70 cell viability was assessed using Prestoblue® assay: PS70 cells were treated with various concentrations of Honokiol (0.5, 1, 2, 5, 10 and 20μM) for 24 and 48 hours. The control cells were treated with DMSO. As shown in (Fig 1A), when exposed to Honokiol concentrations of up to 10μM, there was no statistically significant change in the viability of PS70 cells as compared to the control (n = 12: p < 0.05). However, a significant decrease in cell viability was observed with 20μM Honokiol treatment (n = 12: p < 0.05). The microscopic images further validated the above findings by clearly showing (Fig 1B) no cell death up to 10μM concentrations of Honokiol. Similar to the Prestoblue® assay, cells treated with Honokiol (20μM) showed a relatively higher proportion of dead cells as compared to the control. Moreover, there were significant morphological changes observed with 20μM treatment. Honokiol (20μM) induced extensive shrinkage and fragmentation of PS70 cells, suggesting extensive cell death. Interestingly, we found both time dependent and concentration dependent effects of Honokiol on cell viability. Consequently, based on the results obtained, two highest concentrations of Honokiol (5 and 10μM) at which no cell death was noted were used in the subsequent experiments to elucidate the molecular mechanisms of Honokiol.

### Honokiol increases the activity of antioxidant enzymes in PS70 cells

**Fig 2:**
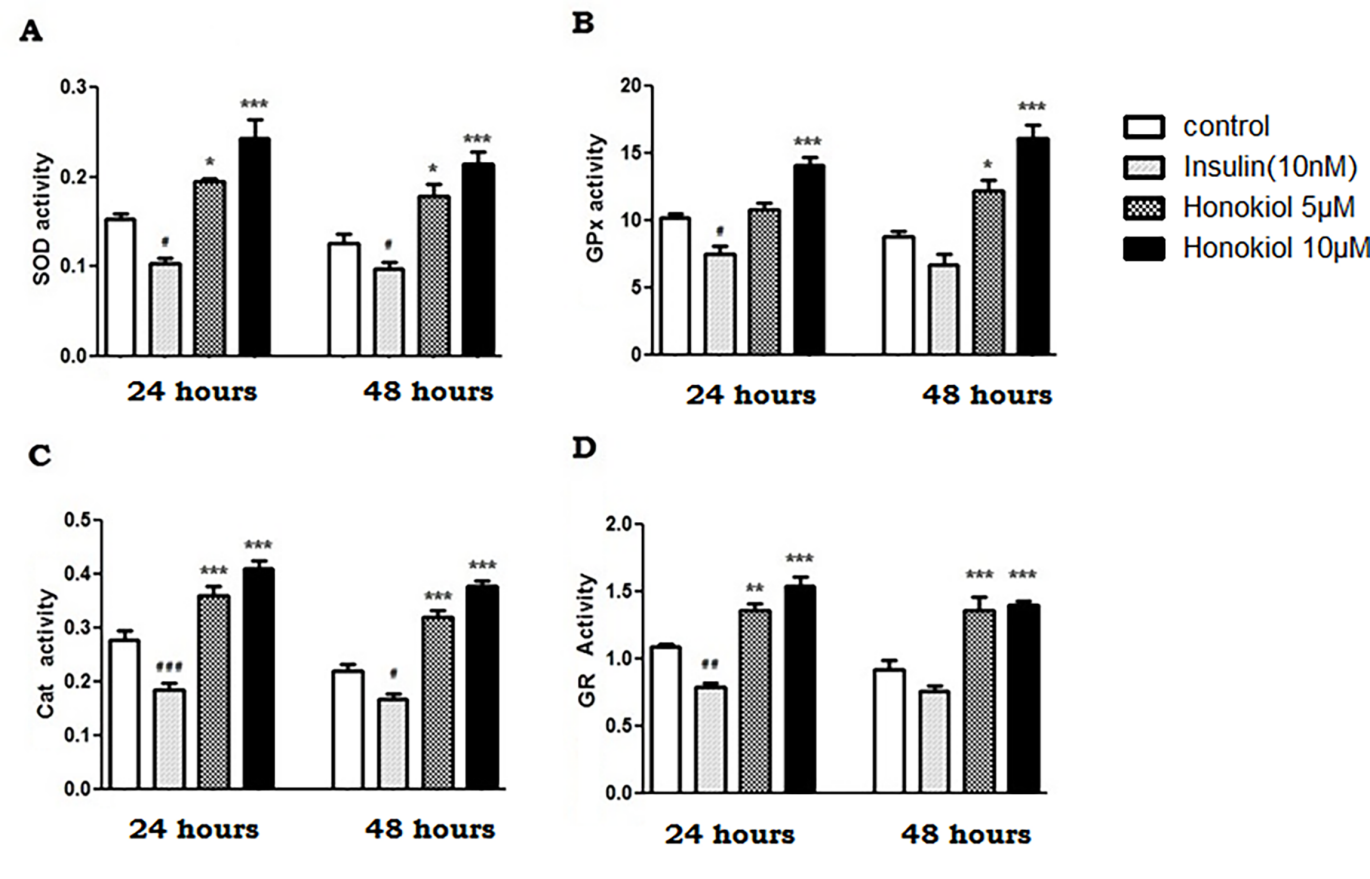
Honokiol increases antioxidant enzymes on PS70 cells. Effect of Honokiol (5 and 10 μM) and Insulin (10nM) on the activities of the antioxidant enzymes were assessed on PS70 cells over a 24 and 48 hours. Activity of (A) SOD, (B) GPx (C) CAT and (D) GR activity were determined as described above. The results are expressed as mean ± SEM (n = 6, #/*p<0.1, ##/**p<0.01, and ###/***p< 0.001 compared with the control). Data with multiple comparisons were analyzed using ANOVA with Dunnett's Multiple Comparison Test. [SOD, superoxide dismutase; GPx, glutathione-peroxidase; CAT, Catalase; GR, Glutathione Reductase].

A defense mechanism of the cell is to promote antioxidant expression and activity, which protects against highly reactive oxy or nitro radicals and their harmful toxic effects. We therefore investigated the effect of Honokiol on the activities of superoxide dismutase (SOD), glutathione peroxidase (GPX), catalase (CAT) and glutathione reductase (GR). These are the key antioxidant enzymes that play a significant role in scavenging toxic free radicals. Honokiol (5 and 10μM) significantly increased the activity of SOD (1.2 and 1.6- fold, Fig 2A), GPX (1.1 and 1.4-fold, Fig 2B), CAT (1.3 and 1.5-fold, Fig 2C) and GRX (1.2 and 1.4-fold, Fig 2D) as compared to the control at 24 hours (n = 6, p < 0.05). Honokiol had similar effect on the activity of antioxidant enzymes at 48 hours. Insulin (10nM) significantly decreased the activity of SOD, CAT, GPX and GRX at 24 hours (Fig 2A–2D, n = 6, p < 0.05). At 48 hours, Insulin (10nM) significantly decreased the activity of SOD, and CAT only (Fig 2A–2D, n = 6, p < 0.05).

### Honokiol scavenges reactive oxygen species and inhibits lipid peroxidation in PS70 cells

**Fig 3:**
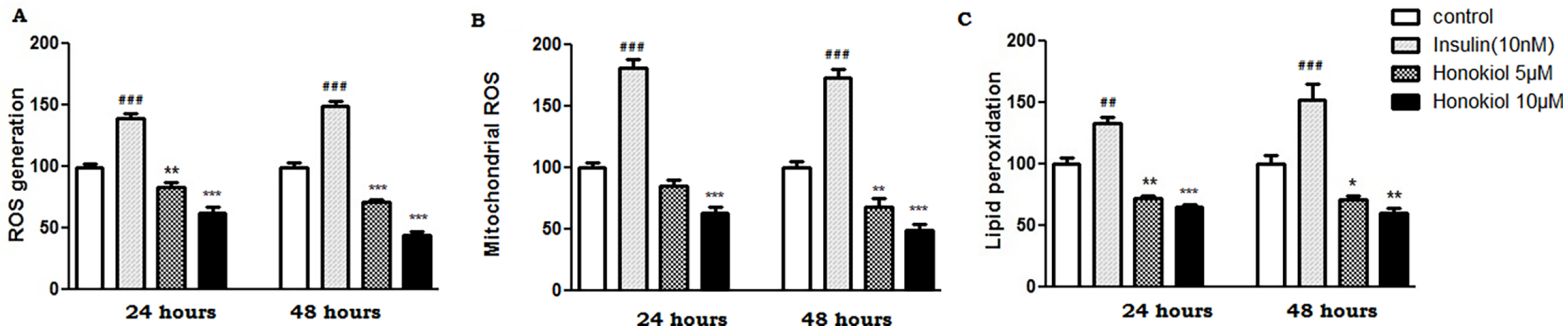
Honokiol scavenges ROS and inhibits lipid peroxidation in PS70 cells. PS70 cells were incubated with Insulin (10nM) and Honokiol (5 and 10 μM). ROS generation was assayed using DCF dye and measured with a spectrophotometer (A) and mitochondrial ROS was measured using DHR assay(B). Lipid peroxidation was measured with a spectrophotometer using the TBARS method (C). The results are expressed as mean ± SEM (#/*p<0.1, ##/**p<0.01, and ###/***p< 0.001 compared with the control). Data with multiple comparisons were analyzed using ANOVA with Dunnett's Multiple Comparison Test (n = 6).

The generation of reactive oxygen species (ROS) triggers oxidative stress and induces irreversible oxidation of lipids and proteins, which has lethal effects on cells viability leading to cell death. Therefore, ROS-induced lipid peroxidation was also investigated in the present study. With respect to the DCF based ROS assay, Honokiol (5 and 10μM) significantly decreased the ROS generation at 24 hours by (14% and 40%) and 48 hours by (29% and 56%) as compared to the control (Fig 3A, n = 6, p <0.05).Insulin (10nM) significantly increased the generation of ROS (43% and 52%) at both the time point (Fig 3A, n = 6, p < 0.05). DHR fluorescent dye was used to further validate the effect of Honokiol and insulin on ROS generation. DHR fluorescent assay yielded similar results as compared to the DCF assay on the ROS generation (Fig 3B, n = 6, p < 0.05). Due to the increase in ROS at 24 and 48 hours, insulin (10nM) significantly increased lipid peroxidation (33 and 52%) as compared to the control (Fig 3C, n = 6, p < 0.05). Since, Honokiol (5 and 10μM) significantly scavenged the ROS; it resulted in decreased lipid peroxide formation by (29% and 36%) at 24 hours and by (30% and 40%) at 48 hours (Fig 3C, n = 6, p < 0.05).

### Honokiol improves mitochondrial bioenergetics

**Fig 4:**
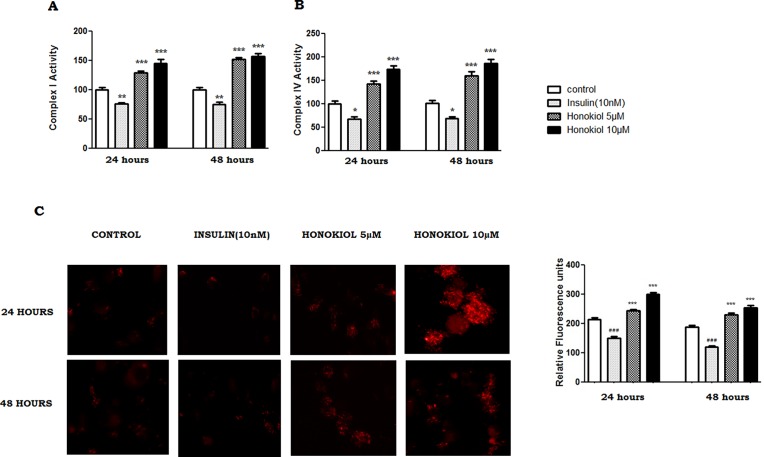
Honokiol improve mitochondrial bioenergetics. Cell lysate analyzed for activities of mitochondrial electron transport chain complex -I and Complex IV activity. The specific activities of complexes I and IV were normalized with respect to specific activities in their corresponding control groups (A and B). All samples are averages ± SEM (n = 6) and (**p<0.01, and ***p< 0.001 compared with the control. Mitochondrial membrane potential measured at 24 and 48 hours (C) following staining of cells with TMRE dye and detected using a fluorescence microscope. Magnification, ×20. Average fluorescence intensity.

To explore the effects of Honokiol on mitochondrial bioenergetics and to understand the molecular processes of mitochondrial function, we evaluated the effects of Honokiol on Complex-I, Complex-IV activity and mitochondrial membrane potential. Honokiol (5 and 10μM) notably improved mitochondrial bioenergetics, as demonstrated by significant increase in Complex-I (29% and 45%) and Complex-IV (43% and 74%) at 24 hours. Similar results with Complex I (52% and 57%) and with Complex IV (61% and 86%) were noted at 48 hours (Fig 4*A* and 4*B*, n = 6, p < 0.05). Likewise, Honokiol (5 μM and 10μM) also increased the mitochondrial membrane potential significantly (13% and 39%) at 24 hours and (21% and 35%) at 48 hours. (Fig 4*C*, n = 6, p < 0.05). Insulin (10nM) significantly inhibited Complex-I activity at 24 and 48 hours (24% and 25%) and Complex-IV activity by (32% and 33%) (Fig 4*A–*4*C*, n = 6, p < 0.05). Similar results were noted with insulin (10nM) on MMP activity at both 24 and 48 hours.

### Honokiol treatment reduces Aβ secretion

**Fig 5:**
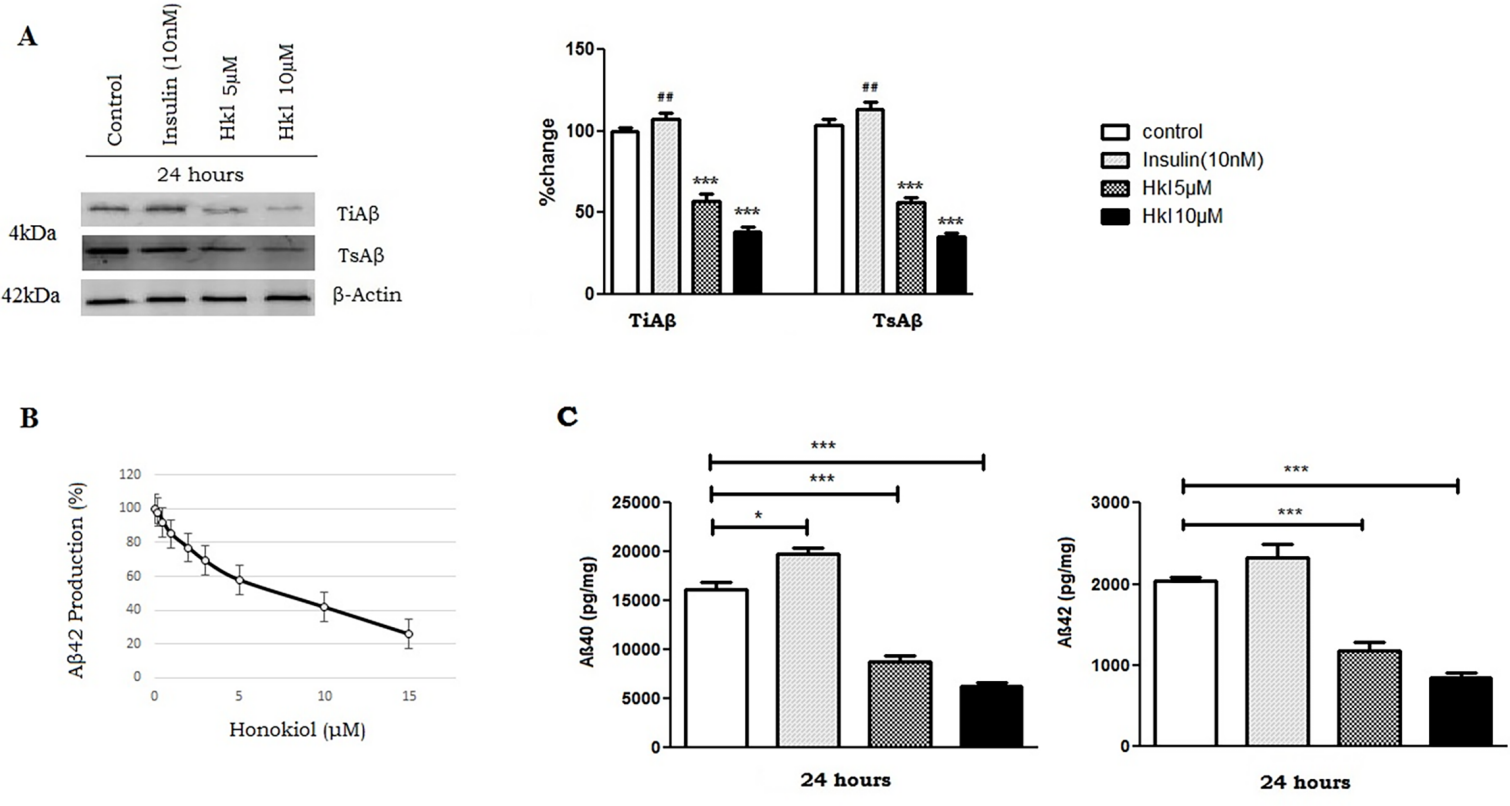
Honokiol treatment reduced Aβ secretion. PS70 cells were treated with DMSO (negative control), Insulin (10nM) or indicated doses of Honokiol for 24h. (A) TsAβ (Total secreted Aβ) and TiAβ (total intracellular Aβ) levels were then analyzed by WB using respective antibodies. The Western blots shown are representative of at least three independent experiments. Densitometric quantification was performed and expressed as percentage change. (B) Concentration response curve of Honokiol shows a dose dependent reduction of Aβ42 production by Honokiol. (C) ELISA measurements of secreted Aβ40 and Aβ42 in conditioned-medium collected from DMSO, Insulin and Honokiol treated PS70 cells. The Aβ results are represented as the mean±SEM of nanograms of Aβ40 or Aβ42 normalized to the amount of total protein [mg] extracted from the cells in the corresponding well. These results are representative of four independent experiments with *n* = 3 for each condition. (One-way ANOVA followed by Dunnett’s post hoc test, n = 3, **: p< 0.01, ***: p< 0.001).

To study whether Honokiol can affect Aβ generation, we measured total Aβ levels by western blot and the results showed that Honokiol (5 and 10μM) significantly decreased total intracellular Aβ (48% and 61%) at 24 hours (Fig 5A). At the same concentration range, Honokiol also reduced total secreted Aβ. Insulin (10nM) showed significantly increased levels of Amyloid-beta levels compared to control. Next, we performed the concentration-response effect of Honokiol (0, 0.2, 0.5, 1, 2, 5, 10 and 15μM) on the generation of Aβ-42. As shown in Fig 5B, there was a dose-dependent decrease in the generation of Aβ-42 in the media by Honokiol. Lower doses (0.1–1μM) of Honokiol had no significant effect. However, there was a significant decrease at 2μM (28%) and a robust decrease at 5 and 10μM Honokiol (40% and 60% respectively). These results clearly confirm that Honokiol can dose dependently decrease Aβ-42 production in this cell-based model. Furthermore, to validate our findings, previous other studies using Honokiol have shown the cytoprotective and neuroprotective effects at 5 and 10μM dose [63]. When PS70 cells were treated with Honokiol (5 and 10μM) for 24 h, levels of Aβ40 and Aβ42 (Fig 5C) in conditioned media were markedly decreased in a dose-dependent manner. On the contrary, Insulin 10nM increased the levels of both Aβ40 and Aβ42 respectively.

### Honokiol modulates amyloidogenic pathway

**Fig 6:**
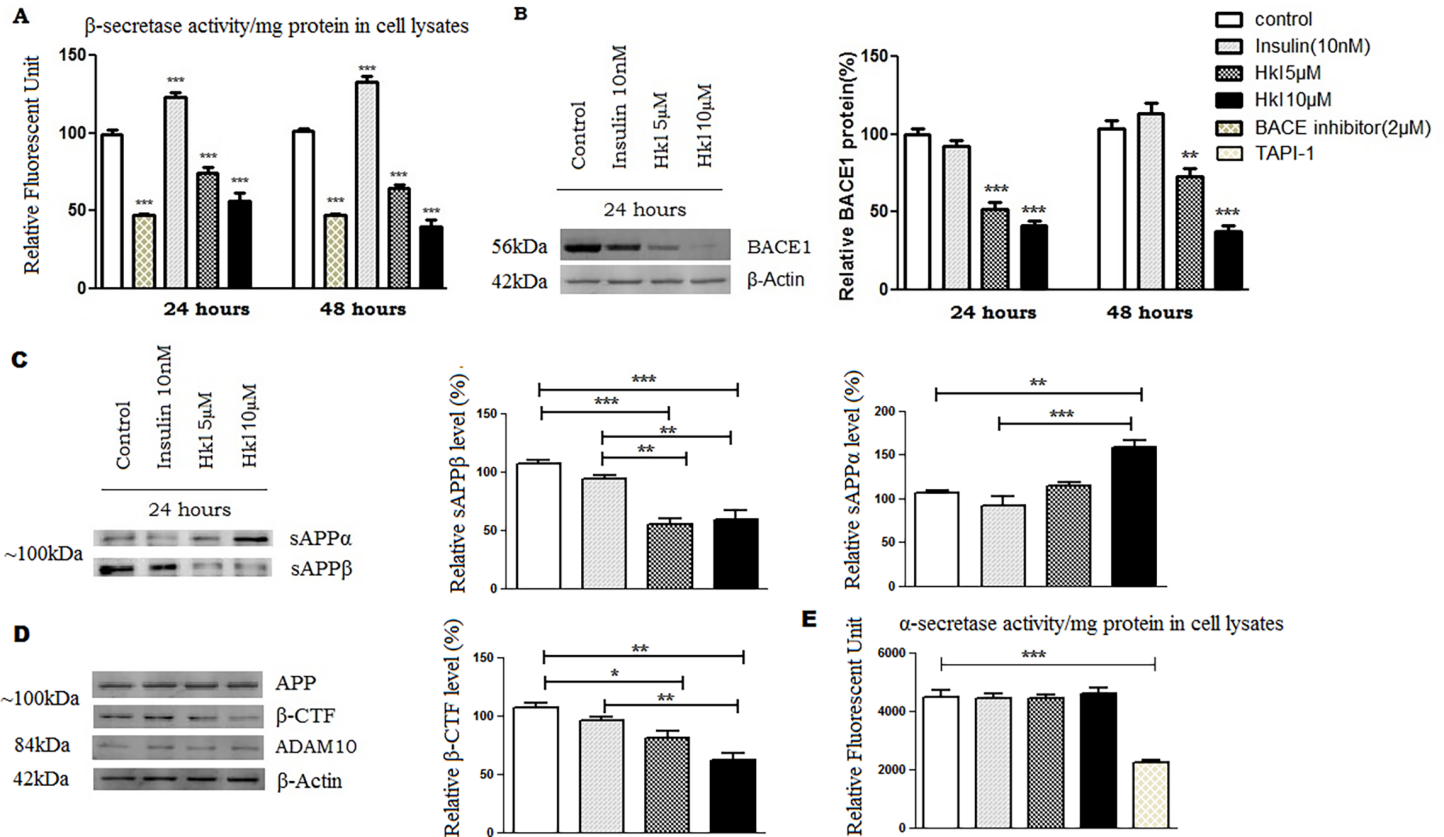
Honokiol treatment reduces amyloidogenic pathway by inhibits β-secretase activity and reducing APP β-CTF and sAPPβ levels. PS70 cells were treated with DMSO (negative control), Insulin (10nM) or indicated doses of Honokiol for 24h. (A) Cell lysates were assayed for β-secretase activity by using a commercial kit from Biovision and subjected to comparison. (B) Cell lysates were processed and examined for BACE expression in Western blots with anti-BACE1 antibodies. β-Actin was used as a loading control. (C) Conditioned media and (D) cell lysates were analyzed by WB using respective antibodies. The Western blots shown are representative of at least three independent experiments. Densitometric quantification was performed and expressed as percentage change. (E) PS70 cells treated with DMSO, insulin 10nM, Honokiol (5 & 10μM) and α-secretase inhibitor TAPI-1 (10μM) for 24 hours. Cell lysates were assayed for α-secretase activity for comparison (One-way ANOVA followed by Dunnett’s post hoc test, n = 3, *: p<0.05, **: p< 0.01, ***: p< 0.001).

Because β-secretase-mediated APP processing is the first step leading to Aβ generation, we studied whether Honokiol affects β-secretase. To ascertain this possibility, we carried out a cell-based assay to measure the β-secretase activity and found that Honokiol dramatically inhibited β-secretase activity by (26% and 44%) at 24 hours and (37% and 60%) at 48 hours (Fig 6A).These results indicate that Honokiol at 10μM exhibited IC50 activity towards β-secretase. Western Blot analysis to detect protein expression showed significantly decreased expression of β-secretase at both the doses (Fig 6B). Since there is another possibility that Honokiol inhibits APP β-processing and Aβ generation through promoting α-secretase activity, we also assayed the activity of TACE, a major α-secretase in PS70 cells treated with Honokiol. We found that Honokiol did not affect TACE activity (Fig 6E), suggesting that Honokiol does not affect α-secretase activity. Honokiol treatment dose-dependently decreased the secreted level of sAPPβ, an amino-terminal fragment of APP generated by β-secretase cleavage by 48% and 40% respectively (Fig 6C). Consistently, the level of APP β-CTF (a carboxyl-terminal fragment of APP generated by β-secretase cleavage) was also decreased upon Honokiol treatment by 23% and 40% (Fig 6D). These results suggest that Honokiol inhibits β-cleavage of APP. In addition, Honokiol (10μM) increased the level of secreted sAPPα, the major extracellular fragment of APP released by α-secretase cleavage (Fig 6C). Moreover, we found that Honokiol treatment did not affect protein levels of APP, and α-secretase ADAM10 (Fig 6D). These results suggest that Honokiol reduces APP amyloidogenic processing not through affecting α but through β-secretase levels [64].

### Honokiol increases SIRT3 and activates AMPK-CREB-PGC1α pathway

**Fig 7:**
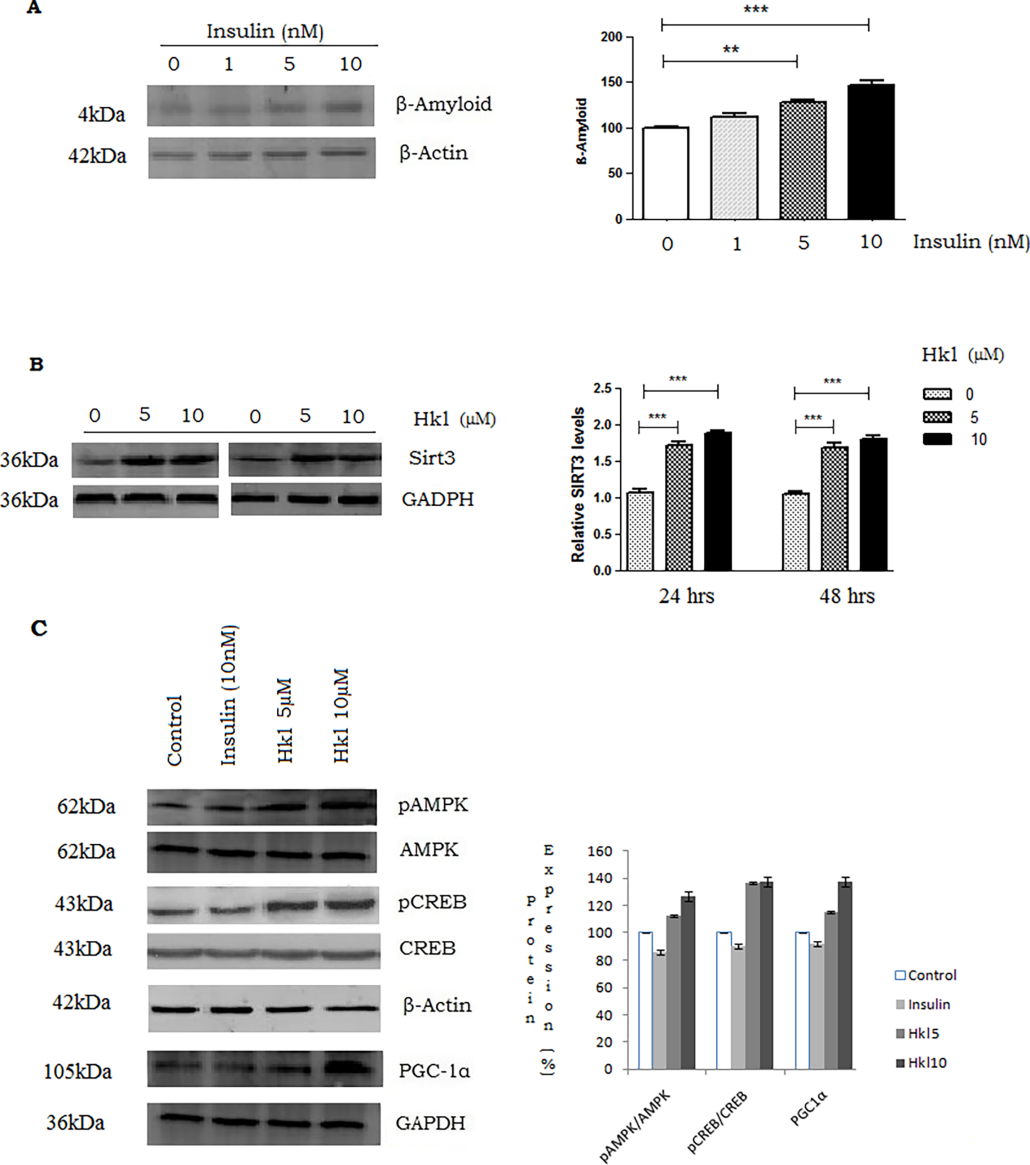
Effect of Honokiol on SIRT3 activation and AMPK-CREB-PGC1α pathway. Effect of increasing concentrations of insulin (0, 1, 5 and 10nM) on TsAβ (Total secreted Aβ) along with the densitometric analysis of band intensity normalized to β-Actin. (B) Representative Western blot of SIRT3 performed on whole cell lysates from PS70 cells exposed to either vehicle (DMSO) or 5 and 10μM Honokiol for 24 and 48 h. The graph displays the densitometric analysis of band intensity of the SIRT3 normalized to the corresponding GADPH level, used as loading control. (C) Effect of Insulin 10nM and Honokiol (5 and 10μM) on p-AMPK/AMPK ratio. Representative Western blot of total AMPK and phosphorylated-AMPK (p-AMPK) levels, total CREB and phosphorylated-CREB (p-CREB) levels and PGC-1α performed on whole cell lysates from PS70 cells exposed to either vehicle, Insulin or Honokiol for 24 h. The graph displays the statistical analysis of the p-AMPK/AMPK and p-CREB/CREB ratio calculated by densitometric analysis of band intensity normalized to the corresponding β-Actin used as loading control; PGC-1α normalized to corresponding GAPDH level, used as loading control. Data, means ± SEM are expressed as percentage of vehicle-treated control; n = 3 under each condition. Significance was calculated with Student's *t* test, *p < 0.05, vs. vehicle-treated cells.

To confirm that high insulin levels predispose to increased Aβ formation, we used increasing concentrations of Insulin (0, 1, 5 and 10nM) on PS70 cells and found that Aβ levels (normalized to β-actin) increased significantly (1.12-, 1.28- and 1.46-fold) compared to control (Fig 7A). Both doses (5 and 10μM) of Honokiol increased SIRT3 levels by nearly twofold and optimum SIRT3 activation was found to be at 24h (Fig 7B). Furthermore, we explored the molecular signaling pathway related to the reduction of Aβ levels by Honokiol. Honokiol (5μMand 10μM) increased the phosphorylation of AMPK by (1.37- and 1.5- fold) compared to total AMPK at 24 hours. Similarly, phosphorylation of CREB was increased by (1.42- and 1.61-fold) with respect to total CREB (Fig 7C). These phosphorylation changes of AMPK and CREB in turn are found to increase the levels of PGC1α. Similarly, we found a statistically significant increase in the levels of PGC1α (1.75) fold at 10μM normalized to GADPH, but no effect was noticed at 5μM (Fig 7C, n = 3, p < 0.05). Insulin (10nM) decreased but did not show a statistically significant change in the phosphorylation of AMPK, CREB and PGC1α.

## Discussion

In this study, we report that Honokiol, an activator of SIRT3 attenuated oxidative stress and beta amyloid secretion in PS70 cells, in addition to improving mitochondrial function. High dose insulin treatment caused increased ROS levels, decrease in mitochondrial functions and increased formation of beta amyloid. Honokiol counteracted these effects by activating SIRT3 and by increasing in AMPK, CREB and PGC1α protein levels thereby causing reduction in beta-secretase activity. Compelling evidence has shown that Honokiol, a SIRT3 activator, expresses many beneficial effects in neurodegenerative diseases [65]. However, there are very few studies that have elucidated the novel mechanisms of SIRT3-mediated decrease in Aβ production. To the best of our knowledge this is the first report describing an activator of SIRT3 capable of improving mitochondrial function, and blocking the beta secretase activity thereby decreasing beta amyloid secretion.

Insulin resistance (IR) is an important risk factor for Alzheimer’s disease and causes an increase in age-related memory impairment [66, 67]. Presence of insulin receptors in the hippocampus and the medial temporal cortex indicate that insulin is known to influence memory and learning [68]. Optimal cerebral insulin levels augment memory and synaptic plasticity in the hippocampus and areneuroprotective [68, 69]. On the contrary, insulin resistance characterized by high insulin levels has been associated with increased levels of reactive oxygen species [70]. High insulin levels promote increased Aβ deposition and tau protein phosphorylation [71–73]. In this study, we further validated the cellular effects of hyperinsulinemia on cognitive impairment. High dose insulin, representing IR, showed an increase in ROS and lipid peroxidation; decrease in the activity of antioxidants; decrease in phosphorylated AMPK, pCREB, and PGC-1α expression. These deficits resulted in decreased mitochondrial functions, increased BACE, and increased Aβ in the PS70 cells. Thus, our results concur with the existing literature showing that hyperinsulinemia can enhance Aβ production.

Oxidative damage has been known to occur at a very early stage of Alzheimer’s disease even prior to Aβ plaque formation and the onset of symptoms [74–76]. Several cellular changes caused by oxidative stress have been related to Aβ plaque formation and pathophysiological events of Alzheimer’s disease [77]. Increased ROS occurs due to an imbalance between pro-oxidants (ROS, RNS, superoxide anion, hydroxyl radicals, and hydrogen peroxide) and antioxidants (GSH, GPX, CAT, GRx, SOD). In addition, ROS leads to deficits in membrane integrity, oxidation of mitochondrial proteins, damage to the mitochondrial respiratory chain, changes in mitochondrial membrane permeability and structure and increased permeability of the plasma membrane to Ca2+[78]. Down-regulation in antioxidant defense mechanisms and elevated ROS generation leads to oxidative stress-mediated neurodegeneration [79].

Exposure of polyunsaturated fatty acids to ROS leads to the production of toxic lipid peroxidation products. Similarly, an increase in the levels of lipid peroxidation was observed in Aβ-induced rat hippocampal cells, due to depletion of antioxidants and increased pro-oxidants [80]. Furthermore, Honokiol has been shown to exert beneficial effects on Aβ-induced toxicity in PC12 cells by inhibiting oxidative stress through reduction of ROS production, intracellular Ca2+ elevation, and caspase-3 activity [81]. In this study, we have reported that Honokiol treatment significantly increased enzymatic antioxidant activities, decreased ROS generation, and decreased lipid peroxidation in PS70 cells. Oxidative stress subsequently leads to impairment of mitochondrial dysfunction [82], which leads to Aβ formation and Aβ induced neurotoxicity [83–86]. At the mitochondrial level, complex I and complex IV seem to be specifically targeted; tau pathology mainly impairs complex I activity and Aβ impairs complex IV activity [87]. Importantly, mitochondrial dysfunction and reduced bioenergetics occur early in pathogenesis and precede the development of plaque formation [88]. Interestingly, hyperinsulinemia has also shown to decrease mitochondrial functions [89]. Our results showed that Honokiol increases the activities of Complex I and IV and increased the mitochondrial membrane potential thereby indicating that it enhances the mitochondrial function.

Furthermore, AMPK is a kinase considered to be a metabolic sensor which is implicated in the regulation of IR and Aβ pathology [90]. Evidence shows that activation of AMPK decreases the production levels of Aβ and AMPK activators like resveratrol have been shown to increase the lysosomal clearance of Aβ [91, 92]. In addition, AMPK enhances mitochondrial biogenesis by inducing PGC-1α transcription and by phosphorylating PGC-1α at threonine-177 and serine-538 [93]. This increased PGC-1α has been shown to decrease BACE and Aβ production. Honokiol increased the phosphorylation of AMPK in a dose-dependent manner and in the same concentration range, increased the phosphorylation of CREB. Together, these results indicate that one of the primary effects of Honokiol is to target AMPK to increase its phosphorylation at Thr-172 and to promote its activation. Furthermore, downstream of AMPK, there is increased phosphorylation of CREB which promotes the activation of PGC-1α. In turn, PGC-1α reduces the activity of β-secretase; reducing Aβ generation through a PPAR-γ-dependent mechanism [94, 95]. Alternatively, SIRT3 is known to directly up regulate the expression of PGC-1α, which increases SIRT3 gene expression [96]. In our study, Honokiol increased the protein levels of AMPK, CREB and PGC-1α thereby decreasing Aβ.

Interestingly, Honokiol had a major role in modulating amyloidogenic pathway. Honokiol had no effect on total APP levels, protein levels of α-secretase ADAM10 and cell based TACE activity, indicating that Honokiol does not affect α-secretase. In contrast, Honokiol treatment decreased protein levels of β-secretase BACE1 and reduced BACE1 enzyme activity, as well as both sAPPβ and APP β–CTF levels, indicating that Honokiol reduces Aβ generation probably through inhibiting β-secretase activity. Hence, we found a modest increase in sAPPα release. Since, γ-secretase complex is part of downstream signaling of both amyloidogenic and non-amyloidogenic pathway, we did not investigate the effect of Honokiol on γ-secretase.

Together, our results demonstrate that Honokiol can reduce Aβ generation in vitro thereby opening avenues for it to be a lead compound for AD drug development.

## Conclusion

**Fig 8:**
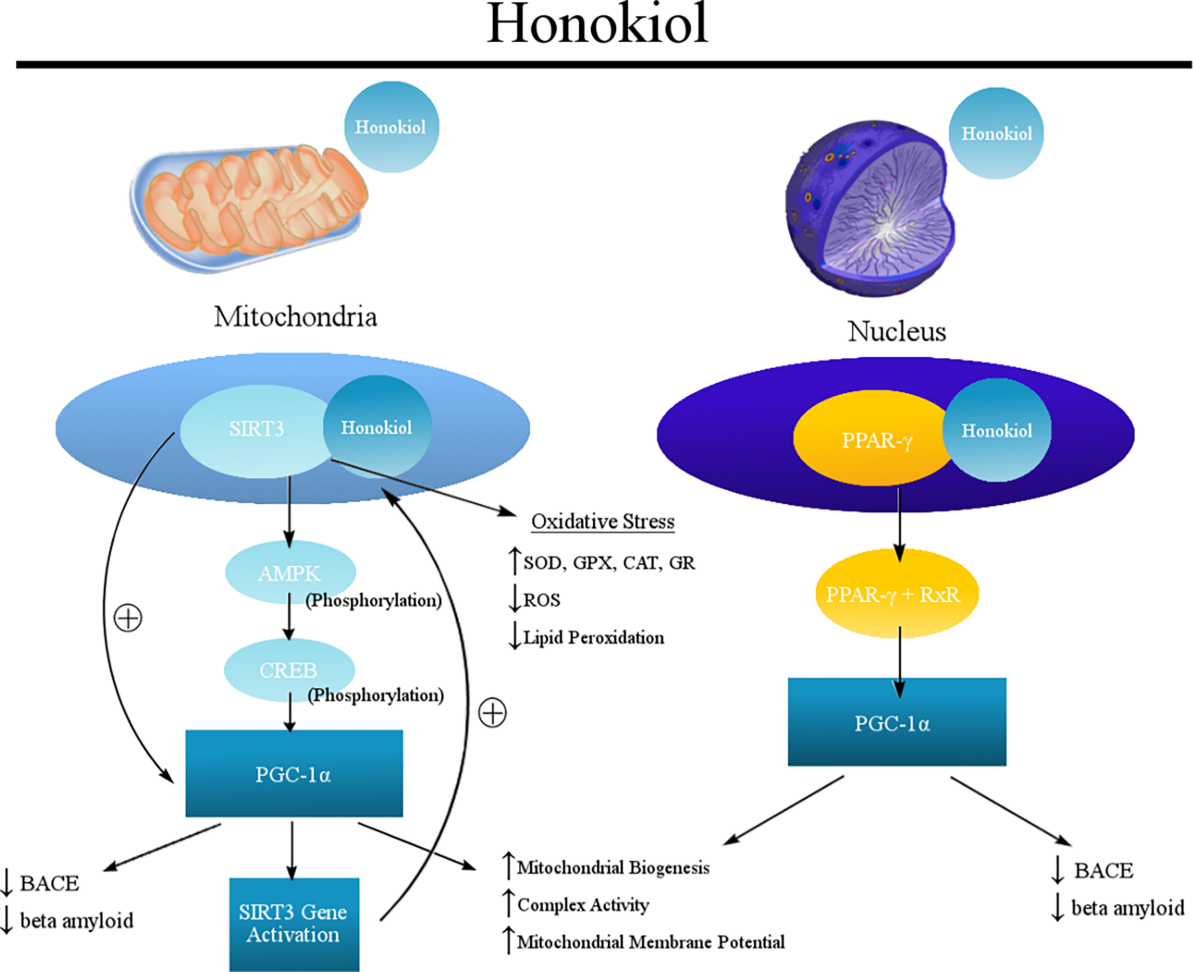
Honokiol mechanism of action.

Honokiol, a dual SIRT3 activator and PPAR-γ agonist, attenuated the markers of oxidative stress, improved cellular antioxidant defense systems, and altered the AMPK pathway, leading to enhanced mitochondrial functions thereby having a modulatory effect on amyloidogenic pathway and eventually decreasing Aβ levels (Fig 8). Overall, these findings demonstrate a potential mitochondrial protective and Aβ reducing effect of Honokiol in PS70 cells. This mechanistic study of Honokiol to suppress pro-oxidative pathways, improve mitochondrial function, and reduce Aβ production prompts further in vitro studies on neuronal cell lines and *in vivo* studies to elucidate the neuroprotective effects of Honokiol in AD. Identifying these functions of Honokiol and their relations to AD will give rise to therapeutic avenues where new concepts can be developed to find an effective treatment.

In the mitochondria, Honokiol binds to SIRT3 and increases the level of SIRT3 through a positive feedback mechanism. Increased levels of SIRT3 enhances mitochondrial biogenesis thereby promoting mitochondrial function and attenuates Amyloid beta levels by acting through AMP-CREB-PGC1α pathway. In the nucleus, increased PGC1α levels promote mitochondrial biogenesis and attenuate amyloid beta levels.

## Abbreviations

Aβ: Amyloid Beta
AD: Alzheimer’s disease
AMPK: 5' adenosine monophosphate-activated protein kinase
APP: Amyloid Precursor Protein
BACE1: Beta or Beta-App Cleaving Enzyme
CAT: Catalase
CHO: Chinese Hamster Ovary
CREB: Camp Response Element-Binding Protein
GPX: Glutathione Peroxidase
GR: Glutathione Reductase
IDE: Insulin Degrading Enzyme
IR: Insulin Resistance
PGC-1α: Peroxisome Proliferator- Activated Receptor Gamma
SOD: Super Oxide Dismutase
ROS: Reactive Oxygen Species
RNS: Reactive Nitrogen Species
